# Healthcare Students and Medical Residents as Second Victims: A Cross-Sectional Study

**DOI:** 10.3390/ijerph191912218

**Published:** 2022-09-26

**Authors:** Carmela Rinaldi, Matteo Ratti, Sophia Russotto, Deborah Seys, Kris Vanhaecht, Massimiliano Panella

**Affiliations:** 1 Department of Translational Medicine (DiMeT), Università del Piemonte Orientale, 28100 Novara, Italy; 2 Learning and Research Area, AOU Maggiore della Carità, 28100 Novara, Italy; 3 KU Leuven Institute for Healthcare Policy, 3000 Leuven, Belgium

**Keywords:** second victim (SV), healthcare students, patient safety incident (PSI), healthcare worker mental health

## Abstract

Background: The term second victim (SV) describes healthcare professionals who remain traumatized after being involved in a patient safety incident (PSI). They can experience various emotional, psychological, and physical symptoms. The phenomenon is quite common; it has been estimated that half of hospital workers will be an SV at least once in their career. Because recent literature has reported high prevalence (>30%) among nursing students, we studied the phenomenon among the whole population of healthcare students. Methods: We conducted a cross-sectional study with an online questionnaire among nursing students, medical students, and resident physicians at the teaching hospital of the University of the Piemonte Orientale located in Novara, Italy. The study included 387 individuals: 128 nursing students, 174 medical students, and 85 residents. Results: We observed an overall PSI prevalence rate of 25.58% (lowest in medical students, 14.37%; highest in residents, 43.53%). Of these, 62.63% experienced symptoms typical of an SV. The most common temporary symptom was the feeling of working badly (51.52%), whereas the most common lasting symptom was hypervigilance (51.52%). Notably, none of the resident physicians involved in a PSI spoke to the patient or the patient’s relatives. Conclusion: Our findings highlighted the risk incurred by healthcare students of becoming an SV, with a possible significant impact on their future professional and personal lives. Therefore, we suggest that academic institutions should play a more proactive role in providing support to those involved in a PSI.

## 1. Introduction

A patient safety incident (PSI) is defined as “an event or circumstance that could have resulted or did result, in unnecessary harm to a patient” [1]. The term “second victim” (SV) describes healthcare professionals who remain traumatized after being involved in a PSI [2]. In fact, they can experience various emotional, psychological, and physical symptoms. Being an SV may lead to dysfunctional coping strategies [3,4], resulting in a change in work behavior and leading to psychological and psychosomatic symptoms such as troubling memory, anxiety, anger, regret, remorse, guilt, and sleeping difficulties [5]. These consequences may superimpose on the depression symptoms a healthcare student may already experience: a recent systematic review showed a prevalence of depression or depressive symptoms of 27.2% among medical students and 11.2% of suicidal ideation [6]. Furthermore, the care of future patients can be negatively affected, leading to defensive changes and reducing the quality of care [7,8]. The phenomenon is quite common. It has been estimated that half of all hospital workers will be an SV at least once in their career [9,10,11]. Recently, further studies suggested that PSIs can occur also in health profession students [12,13]. A systematic review [14] found that 28–30% of nursing students were involved in medication errors during their clinical practice, while in Italy, a PSI density of 3.8/1000 days of clinical training was observed [15]. Therefore, as well as healthcare workers, students and medical residents are likely to be exposed both directly and indirectly to PSIs during their clinical practice, and the lack of adequate support may have a detrimental impact on their professional identity, values, and expectations. Moreover, understanding students’ physical and psychological responses to PSIs can raise awareness and interest in the SV phenomenon and can be used to develop coping abilities, helping them to adapt to the clinical field when they become healthcare professionals. Resident physicians also seem to be a particularly susceptible category of healthcare worker, similar to students (in Italy, the medical specialization is an academic course, whereas in most countries it is not). In fact, a recent study found that 88% of surgical residents felt part of a medical error and 86.5% of those experienced subsequent emotional sequelae, most commonly guilt, anxiety, and insomnia. Moreover, only 24% of them received support [16]. For the above reasons, we performed a study aimed at describing the prevalence of SVs among Italian nursing students, medical students, and resident physicians while detecting any possible differences among such groups. As secondary objectives, we investigated the physical and psychological symptoms in the aftermath of a PSI in these groups, analyzing the perceived causes and the received support.

## 2. Materials and Methods

### 2.1. Study Design and Setting

We designed a cross sectional study that used a web-based questionnaire to describe the epidemiology of the SV phenomenon among the students at the School of Medicine. The setting was the University of Eastern Piedmont (UNIUPO) Teaching Hospital “Ospedale Maggiore della Carità”, located in Novara, Italy. The initiative was approved by the academic board of directors of the School of Medicine and the study protocol was approved by the local ethical committee (protocol number 639/CE).

### 2.2. Population

We invited to the research all the students at the School of Medicine of the UNIUPO with at least six months of clinical training experience. This included the students attending the fourth, fifth, and sixth year of the medicine and surgery course, the second and third year of the nursing course, and all the resident physicians attending the clinical and surgical schools of specialization. In total, there were 488 eligible students who were sent the invitation to participate: 91 second-year nursing students, 93 third-year nursing students, 83 third-year medical students, 123 fifth-year medical students, and 98 sixth-year medical students.

### 2.3. Recruitment of Participants

As a kickoff of the study, we held a grand rounds presentation of the initiative for each group of participants. The didactic tutors for the nursing students, the coordinators of the clinical tutors for medicine students, and the directors of the schools of specialization for the resident medical doctors participated in the grand rounds. The presentation included the definition of both a patient safety incident and a second victim, along with the description of the different sections of the questionnaire. We also held a question and answer session at the end of the presentation. After the presentation, all the students received an online URL link through the institutional email address. Upon clicking the link, the participants were firstly prompted by an informed consent and, if accepted, the online questionnaire. The students were also specifically informed about the possibility of resigning at any time, even after completing the survey, by contacting the principal investigator. All the participants were volunteers without financial incentives and respondents were guaranteed anonymity and confidentially according to the European Union General Data Protection Regulation (GDPR EU law 2016/679) and the relevant Italian privacy bylaws.

### 2.4. Sample Size

According to the study design, we based the sample size calculation on the main outcome of the study—being an SV—assuming a prevalence of SV of 30% from literature [17]. The required sample size for a cross sectional survey study at an alpha level of 0.05, an absolute error of 5%, and a power of 0.8 is 377 participants.

### 2.5. Questionnaire

The questionnaire was a web-adapted Italian version of the questionnaire used in previous research targeted to nursing and medicine students [8,18]. The preliminary Italian version of the questionnaire was subject to pre-testing to ensure feasibility and readability. We included 20 volunteer subjects, with characteristics similar to those of the sample involved in the present study. The final questionnaire consisted of a preliminary part of demographic characteristics (age, gender, year of enrollment, months of clinical experience) and a second part of 26 items belonging to three areas. The first area included eight items investigating the prevalence and the setting of PSIs. The second section consisted of five questions that were available only for students who either directly or indirectly encountered a PSI during their clinical training practice. This section investigated the students’ physical and psychological responses to PSI and the resulting effects both at the personal and professional levels. The last section consisted of 13 items investigating post-event support received or expected by the students after a PSI. The average time estimated for completing the entire survey was 10 min. Each closed-ended question allowed multiple preferences; empty fields were provided to answer open-ended questions with characters limitations. By means of a reset button, respondents were also able to review and change their answers. The presence of any missing answers prevented respondents from proceeding with the survey and completeness checks appeared before each page was submitted. The full questionnaire in its original English version is reported in Supplementary Material S1.

### 2.6. Data Collection

Data were collected using an online form implemented in the SoGoSurvey platform (2291 Wood Oak Drive Suite 300, Herndon, VA, USA). The questionnaire was available online for 10 weeks to accommodate student schedule availability. For the same reason, the opportunity to interrupt the survey and resume it at a later time was implemented with unique credentials. The data were anonymized before analysis and presented as aggregate.

### 2.7. Data Analysis

The data collected were processed and analyzed with R v. 4.1.0 (R Foundation for Statistical Computing, Vienna, Austria) and tidyverse package v. 1.3.1. We first tested the distribution of each variable. We described continuous measures with means and standard deviation and tested with the *t*-test if normally distributed; otherwise, we described the variables with median, interquartile range, and the Wilcoxon test. Categorical data was described with frequencies and proportions, and tested with the chi-square or Fisher test. We calculated the prevalence ratio along with its 95% confidence interval in aggregate and for each group of students, comparing the proportion of the characteristics in each group to the whole sample. To avoid misinterpretation due to the different statuses of the resident physicians between countries, we excluded them from the comparative analysis, leaving their data only for descriptive purposes. We set an alpha level of significance of 0.05 and the test was conducted with “two tails”.

## 3. Results

### 3.1. Participants Demographics

In total, 387 responders were included in the study: 128 nursing students (33.1%), 174 medical students (45%), and 85 medical residents (22%). In our sample most of the responders were female (64,85%), with a different distribution among nursing students (80.47%), residents (60%), and medical students (55.75%). The overall mean age was 25.16. The youngest were nursing students (23.09 years), followed by medical students (24.24 years) and residents (29.92 years). These results are summarized in Table 1.

### 3.2. Prevalence of Patient Safety Incidents and Second Victims

As shown in Table 2, 99 subjects (25.58%) had been involved in a PSI during their training. The frequency was significantly lower in medical students and higher among residents (*p* < 0.01). The distribution of PSI-involved individuals between male and female was 35.3% and 64.6%, respectively. We measured an overall prevalence rate of SVs of 16.02%. This was significantly lower among medical students and higher among residents (4.6% and 31.76%, respectively).

### 3.3. The Level of Severity of the Incident

Of the 99 participant who were involved in a PSI, 60 reported that the PSI caused harm to the patient (60.61 %). Of those PSIs, 41 (41.41 %) caused temporary harm to the patient, 6 (6.06 %) caused permanent harm to a patient, and 13 (13.13%) caused the death of the patient. We also observed a significantly higher proportion of residents directly involved in a PSI when compared to the other groups (*p* = 0.046). We did not observe any significant difference among the groups in their opinion on the avoidability of the PSI.

### 3.4. Perceived Causes of PSIs

**Figure 1:**
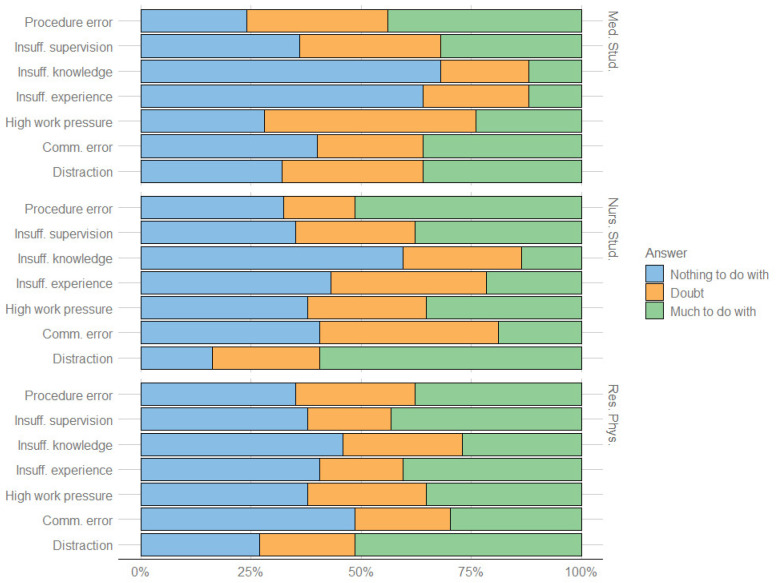
Students’ opinions about the causes of PSIs.

In Figure 1, we described the perceived causes of PSI. Most of the participants considered distraction as the main cause of PSI (50.51%), followed by procedural errors (44.44%), insufficient supervision (38.38%), high work pressure (32.32%), communication errors (27.27%), insufficient experience (26.26%), and insufficient knowledge (18.18%). The only significant difference among the groups concerned insufficient level of experience, which was considered to be the cause of the PSI by the 21.62% of nursing students and 12% of medical students (*p* = 0.03).

### 3.5. Aftermath of a Patient Safety Incident

**Figure 2:**
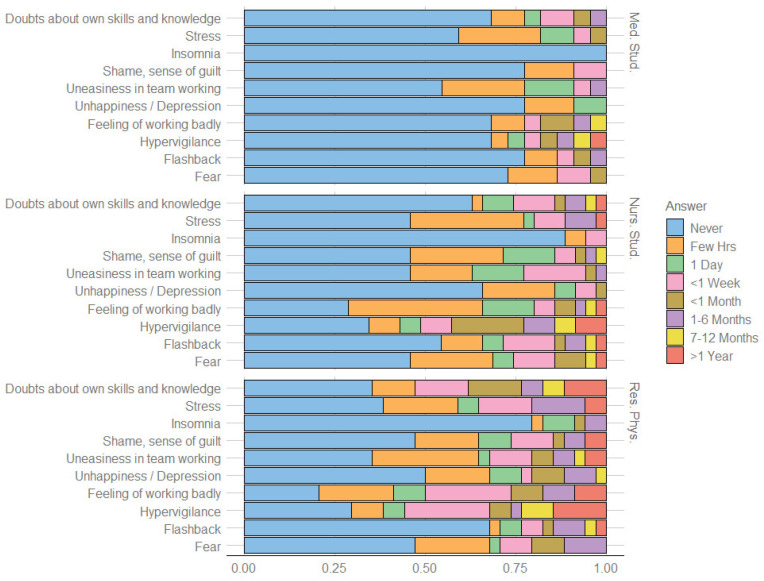
Responders’ duration of symptoms after the PSI.

The 99 participants who were involved in a PSI were asked to describe its impact on their life. Of these students, 62.63% became an SV after the PSI, with a lower probability for medical students (*p* < 0.01) (Table 2). Of participants involved in a PSI, 38.38% experienced at least one lasting symptom (>1 month) and the most frequent of these was hypervigilance (23.23%). Among the temporary symptoms (<1 month), however, the most frequent was the feeling of working badly (51.52%). As can be seen in Figure 2, the temporary feeling of working badly is lower among medical students (24%) than among nursing students (62.16%) and resident physicians (59.46%). Furthermore, none of the students reported experiencing unhappiness or depression for more than a month, unlike the residents (10.81%).

### 3.6. Source of Support towards PSIs

As we can see in Table 3, almost all of the students involved in a PSI talked to someone, mainly to colleagues, followed by friends, clinical tutors, nurses, and their partners. Only few of them spoke to the patients or patient’s relatives. The University Counseling Service (3.03%) and the General Practitioner Clinic (1.01%) were almost unused as sources of support. Medical residents more frequently preferred to speak to their clinical tutors, their teaching tutor, and with physicians. Furthermore, none of the residents involved in a PSI spoke to the patient (0%) or to the patient’s relatives (0%). Nursing students, on the other hand, speak to nurses more frequently than the other group of students (70.27%, *p* < 0.01).

## 4. Discussion

As a major finding, our study showed that more than a quarter of the participants was involved in a PSI, which determined an SV prevalence rate of 16.02%. While the prevalence of PSI was consistent with the scarce available literature, the prevalence of SVs was lower [10,13]. This could be due to the heterogeneity of our sample, which included not only nurses, but also medical students and resident MDs. Regarding PSIs, we observed the lowest prevalence among medical students and the highest prevalence among resident physicians. Medical students dedicate only a fraction of their time to practical training in wards, as they also attend classes; therefore, it is reasonable to think that they were less “exposed” to possible PSIs when compared to the residents. Furthermore, medical student training in wards is mostly observational, while residents are more involved in clinical practice, with higher levels of autonomy and responsibility This could be the reason why we observed that the proportion of resident physicians directly involved in a PSI was higher. We also think that the time spent in direct care of patients may be proportional to the risks of PSI incurrence and of becoming an SV. In fact, medical students have the shorter internship, followed by nursing students and resident physicians, but further research should be conducted to better explain any association between these variables. The prevalence rate of SVs was lower among medical students and higher among resident physicians. While we do not think that medical students were intrinsically more resilient to PSIs than residents, we may attribute this finding to the different level of autonomy in the clinical practice of the two groups. As has been mentioned before, students’ clinical training is more “observational” and, therefore, they may feel less responsible. On the contrary, more experienced workers such as residents tend to suffer more from being an SV, as confirmed by literature [19].

Previous studies have shown that workers permanently changed their attitudes towards their job after being involved in a PSI, for example, adopting defensive practices that could reach 10% of the national health expenditure [20]. Therefore, PSI incurrence at the beginning of careers could result in even higher expenses for national healthcare services, because these costly practices are perpetrated for a long time, up to the entire working life of the individual healthcare worker. Noteworthily, young second victims also deliver lower quality health care to patients for a very extended period.

Almost 20% of the participants reported involvement in a PSI resulting in permanent harm or death of the patient. According to literature, these participants are particularly at risk for poor well-being and reduced professional functioning during their clinical experience from being involved in a PSI with such serious consequences; involvement in a PSI can have a serious impact on mental health, so it is important to recognize early SV symptoms [21,22,23]. The main perceived causes of the reported PSIs (distraction, procedural errors) were consistent with other similar studies [13,18]. It is interesting to note that, in our study, lack of experience as a cause of PSIs was reported by 40% of resident physicians. According to literature, this could suggest that these professionals could be more vulnerable to the effects of PSIs and more likely to become SVs [24]. Furthermore, resident physicians suffered the highest level of unhappiness or depression, which, on average, lasted for more than one month. We think that this adds further evidence that this group of healthcare workers is the most emotionally vulnerable to PSIs [24]. Therefore, we think that residency programs/specialization schools should consider including structured programs for supporting residents after experiencing a PSI. We did not find any relevant data about effective interventions already implemented. However, since these symptoms closely resemble post-traumatic stress disorder (PTSD) [12,25], we think that when defining a potential intervention towards PSIs involving healthcare students, a PTSD program should be considered. As an example, psychological therapy, self-help programs, and even drug therapies may be considered, because of their effectiveness towards PTSD in veterans [26,27].

Regarding the impact of PSIs, the most common symptoms were hypervigilance and the temporary feeling of working badly. Our findings confirmed previous research concerning the symptoms in the aftermath of a PSI [18]. Moreover, we observed that the temporary feeling of working badly was lower among medical students when compared with nursing students. We think that this result could be linked to a biased perception of the activity in the ward in the groups of respondents. Medical students may not perceive their internship as a working activity, unlike nursing students, who are more involved in clinical activities. As a consequence, they are less likely to report the feeling of working badly after a PSI. Seeking support is a common coping strategy for SVs [21]. This was confirmed by our results, where almost all of the participants talked to someone about the PSI, as expected, mainly with their colleagues [28]. Our findings also showed that most of the participants did not talk about the PSI with the patient or the family’s patient. Literature suggests that, despite recommendations on the importance of communicating with patients and their relatives after a PSI, healthcare workers may still fear the consequences of such a conversation [29]. However, we think that, in our sample, this attitude was enhanced by the fact that our respondents did not have direct responsibility for this activity.

### Limitations of the Study

Our study has several limitations, mostly related to the nature of a self-reporting survey [30]. In self-reporting, it is possible that respondents give socially desirable answers. Furthermore, respondents have a ‘blind spot’ in the perception they have about themselves [31]. In addition, the representativeness of the sample can be questioned because not all residency programs/specialization schools participated to the study. We think that this could reduce the strength of our findings and, for these reasons, we believe that it may be useful to carry out further research to better understand PSIs and the SV phenomenon among health profession students.

## 5. Conclusions

Our results reported new data about the prevalence, the symptoms, and the support received by nursing students, medical students, and resident physicians in the aftermath of a PSI. Our findings showed that SVs are common among healthcare profession students. This is an underestimated and undertreated problem, both in hospitals and in academia. Therefore, we think that academic institutions and healthcare organizations should play a more proactive role in providing support to those involved in a PSI and should not wait until students burn-out or quit their education.

## Figures and Tables

**Table 1:** Description of participant demographics.

	Total Sample	Nursing Students	Medical Students	Resident Physicians
Respondents *n*/*n* (%)	387	128/387 (33.1%)	174/387 (45%)	85/387 (22%)
Age, (years, mean)	25.16	23.09	24.24	29.92
Gender (female, *n*/*n*) (%)	251/387 (64.85%)	103/128 (80.5%)	97/174 (55.7%)	51/85 (60%)
Months of traineeship	14.13	8.5	12.5	25.8

**Table 2:** Description of PSI and second victim phenomenon. Statistically significant results are highlighted in **bold**.

	Total Responders (%)	Nurs. Stud. (%–95%CI)	Med. Stud. (%–95%CI)	Res. Phys. * (%)
PSI prevalence rate (*n* = 387, 128, 174, 85)	25.58	28.91 (21.41–37.69)	**14.37 (9.69–20.67 ) ^1^**	43.53
Second victim prevalence rate (*n* = 387, 128, 174, 85)	16.02	21.09 (14.59–29.38)	**4.6 (2.15–9.17) ^2^**	31.76
Impact of PSI experienced (risk of becoming SV) (*n* = 99, 37, 25, 37)	62.63	72.97 (55.61–85.62)	**32 (15.73–53.55) ^3^**	72.97
Of those: Impact on professional life	34.34	40.54 (25.20–57.81)	20 (7.61–41.3)	37.84
Impact on personal life	11.11	16.22 (6.77–32.68)	0 (0–16.57)	13.51
Impact on both	17.17	16.22 (7.03–32.23)	12 (3.15–32.34)	21.62
Duration of symptoms >1 month (*n* = 99, 37, 25, 37)	38.38	37.84 (23.61–54.43)	32 (15.73–53.55)	43.24
Direct participation/contribution to the PSI (*n* = 99, 37, 25, 37)	27.27	24.32 (12.37–41.56)	8 (1.40-27.50)	43.24
Negative consequences of PSI on patient (*n* = 99, 37, 25, 37)	60.61	51.35 (34.67–67.76)	60 (40.17–77.08)	67.57
Of those: temporary lesion	41.41	43.24 (27.50–60.36)	36 (18.71–57.38)	43.24
permanent lesion	6.06	5.41 (1.22–18.60)	4 (0.21–22.32)	8.11
death	13.13	2.7 (0.14–15.81)	24 (10.16–45.52)	16.22
Participants opinion on avoidability of PSI : Yes or easily avoidable (*n* = 99, 37, 25, 37)	84.84	94.59 (80.47–99.06)	76 (54.48–89.84)	81.08

^1^*p* < 0.001, ^2^ *p* < 0.001, ^3^ *p* < 0.01, * Resident physicians were excluded from the statistical comparative analysis.

**Table 3:** Responder behavior after the PSI. Statistically significant results are highlighted in **bold**.

	Total % (*n* = 99)	Nurse Std % (95%CI) (*n* = 37)	Medical Std % (95%CI) (*n* = 25)	Res Physician * (*n* = 37)
Have you ever spoken to someone about the PSI?	95.96	94.59 (80.47–99.06)	96.00 (80.40–99.30)	97.30
Professional Area				
Clinical tutor	49.49	45.95 (29.85–62.87)	32.00 (15.72–53.55)	64.86
Didactic tutor	25.25	16.22 (6.77–32.68)	16.00 (5.25–36.91)	40.54
Professor	12.12	2.70 (0.14–15.81)	4.00 (0.21–22.32)	27.03
University counseling service	3.03	0.00 (0.00–11.71)	4.00 (0.44–20.91)	5.41
University colleagues	76.77	72.97 (55.61–85.63)	80.00 (58.70–92.39)	78.38
Nurses	49.49	**70.27** **(52.83–83.56)**	28.00 (12.87–49.60)	43.24
Head nurse	14.14	13.51 (5.53–28.72)	12.00 (3.15–32.34)	16.22
Physicians	41.41	**21.62** **(10.42–38.66)**	44.00 (25.02–64.73)	59.46
Personal Area				
Family MD	1.01	0.00 (0.00–11.15)	4.00 (0.21–22.32)	0.00
Relatives	42.42	45.95 (29.85–62.87)	48.00 (28.34–68.25)	35.14
Friends	52.53	59.46 (42.19–74.80)	52.00 (33.03–70.42)	45.95
Partner	47.47	45.95 (29.85–62.87)	52.00 (31.76–71.66)	45.95
First Victim Area				
Patient	11.11	16.22 (6.77–32.68)	20.00 (7.61–41.30)	0.00
Patient’s relatives	9.09	13.51 (5.08–29.57)	16.00 (5.25–36.92)	0.00
If you talked to anyone, will you talk again in case of a future PSI?	85.86	89.19 (73.64–96.48)	84.00 (63.24–94.67)	83.78

* Resident physicians were excluded from the statistical comparative analysis.

## Data Availability

Anonymized data is available upon motivated request.

## Supplementary Materials

The following supporting information can be downloaded at: https://www.mdpi.com/article/10.3390/ijerph191912218/s1.

## Abbreviations

The following abbreviations are used in this manuscript:

PSI	Patient Safety Incident
SV	Second Victim

## References

[1] World Health Organization (2010). Conceptual Framework for the International Classification for Patient Safety Version 1.1: Final Technical Report January 2009.

[2] Wu A.W. (2000). Medical error: The second victim. The doctor who makes the mistake needs help too. BMJ (Clin. Res. Ed.).

[3] Busch I.M., Moretti F., Purgato M., Barbui C., Wu A.W., Rimondini M. (2020). Dealing With Adverse Events: A Meta-analysis on Second Victims’ Coping Strategies. J. Patient Saf..

[4] Kappes M., Romero-García M., Delgado-Hito P. (2021). Coping strategies in health care providers as second victims: A systematic review. Int. Nurs. Rev..

[5] Busch I.M., Moretti F., Purgato M., Barbui C., Wu A.W., Rimondini M. (2020). Psychological and Psychosomatic Symptoms of Second Victims of Adverse Events: A Systematic Review and Meta-Analysis. J. Patient Saf..

[6] Rotenstein L.S., Ramos M.A., Torre M., Segal J.B., Peluso M.J., Guille C., Sen S., Mata D.A. (2016). Prevalence of Depression, Depressive Symptoms, and Suicidal Ideation Among Medical Students: A Systematic Review and Meta-Analysis. JAMA.

[7] Liukka M., Steven A., Moreno M.F.V., Sara-Aho A.M., Khakurel J., Pearson P., Turunen H., Tella S. (2020). Action after Adverse Events in Healthcare: An Integrative Literature Review. Int. J. Environ. Res. Public Health.

[8] Panella M., Rinaldi C., Leigheb F., Donnarumma C., Kul S., Vanhaecht K., Di Stanislao F. (2016). The determinants of defensive medicine in Italian hospitals: The impact of being a second victim. Rev. Calid. Asist. Organo Soc. Esp. Calid. Asist..

[9] Mohamadi-Bolbanabad A., Moradi G., Piroozi B., Safari H., Asadi H., Nasseri K., Mohammadi H., Afkhamzadeh A. (2019). The second victims’ experience and related factors among medical staff. Int. J. Workplace Health Manag..

[10] Seys D., Wu A.W., Van Gerven E., Vleugels A., Euwema M., Panella M., Scott S.D., Conway J., Sermeus W., Vanhaecht K. (2013). Health care professionals as second victims after adverse events: A systematic review. Eval. Health Prof..

[11] White R.M., Delacroix R. (2020). Second victim phenomenon: Is ‘just culture’ a reality? An integrative review. Appl. Nurs. Res..

[12] Choi E.Y., Pyo J., Ock M., Lee H. (2021). Second victim phenomenon after patient safety incidents among Korean nursing students: A cross-sectional study. Nurse Educ. Today.

[13] Van Slambrouck L., Verschueren R., Seys D., Bruyneel L., Panella M., Vanhaecht K. (2021). Second victims among baccalaureate nursing students in the aftermath of a patient safety incident: An exploratory cross-sectional study. J. Prof. Nurs. Off. J. Am. Assoc. Coll. Nurs..

[14] Asensi-Vicente J., Jiménez-Ruiz I., Vizcaya-Moreno M.F. (2018). Medication Errors Involving Nursing Students. Nurse Educ..

[15] Stevanin S., Causero G., Zanini A., Bulfone G., Bressan V., Palese A. (2018). Adverse events witnessed by nursing students during clinical learning experiences: Findings from a longitudinal study. Nurs. Health Sci..

[16] Khansa I., Pearson G.D. (2022). Coping and Recovery in Surgical Residents after Adverse Events: The Second Victim Phenomenon. Plast. Reconstr. Surgery. Glob. Open.

[17] Scott S.D., Hirschinger L.E., Cox K.R., McCoig M., Hahn-Cover K., Epperly K.M., Phillips E.C., Hall L.W. (2010). Caring for Our Own: Deploying a Systemwide Second Victim Rapid Response Team. Jt. Comm. J. Qual. Patient Saf..

[18] Vanhaecht K., Seys D., Schouten L., Bruyneel L., Coeckelberghs E., Panella M., Zeeman G., Dutch Peer Support Collaborative Research Group (2019). Duration of second victim symptoms in the aftermath of a patient safety incident and association with the level of patient harm: A cross-sectional study in the Netherlands. BMJ Open.

[19] Fatima S., Soria S., Esteban- Cruciani N. (2021). Medical errors during training: How do residents cope?: A descriptive study. BMC Med. Educ..

[20] Panella M., Rinaldi C., Leigheb F., Knesse S., Donnarumma C., Kul S., Vanhaecht K., Di Stanislao F. (2017). Prevalence and costs of defensive medicine: A national survey of Italian physicians. J. Health Serv. Res. Policy.

[21] Quillivan R.R., Burlison J.D., Browne E.K., Scott S.D., Hoffman J.M. (2016). Patient Safety Culture and the Second Victim Phenomenon: Connecting Culture to Staff Distress in Nurses. Jt. Comm. J. Qual. Patient Saf..

[22] Ulenaers D., Grosemans J., Schrooten W., Bergs J. (2021). Clinical placement experience of nursing students during the COVID-19 pandemic: A cross-sectional study. Nurse Educ. Today.

[23] Vanhaecht K., Seys D., Bruyneel L., Cox B., Kaesemans G., Cloet M., Van Den Broeck K., Cools O., De Witte A., Lowet K. (2021). COVID-19 is having a destructive impact on health-care workers’ mental well-being. Int. J. Qual. Health Care J. Int. Soc. Qual. Health Care.

[24] Torbenson V.E., Riggan K.A., Weaver A.L., Long M.E., Finney R.E., Allyse M.A., Rivera-Chiauzzi E. (2021). Second Victim Experience among OBGYN Trainees: What Is Their Desired Form of Support?. South Med. J..

[25] Ben Saida I., Grira S., Toumi R., Ghodhbani A., Ennouri E., Meddeb K., Ben Saad H., Boussarsar M. (2022). North-African doctors as second victims of medical errors: A cross sectional survey. BMC Psychiatry.

[26] Disorder P.S. (2014). Treatment for Posttraumatic Stress Disorder in Military and Veteran Populations: Final Assessment.

[27] Bisson J.I., Cosgrove S., Lewis C., Roberts N.P. (2015). Post-traumatic stress disorder. BMJ.

[28] Engel K.G., Rosenthal M., Sutcliffe K.M. (2006). Residents’ responses to medical error: Coping, learning, and change. Acad. Med. J. Assoc. Am. Med. Coll..

[29] Mira J.J., Lorenzo S., Carrillo I., Ferrús L., Silvestre C., Astier P., Iglesias-Alonso F., Maderuelo J.A., Pérez-Pérez P., Torijano M.L. (2017). Lessons learned for reducing the negative impact of adverse events on patients, health professionals and healthcare organizations. Int. J. Qual. Health Care J. Int. Soc. Qual. Health Care.

[30] Polit D.F., Beck C.T. (2006). The content validity index: Are you sure you know what’s being reported? Critique and recommendations. Res. Nurs. Health.

[31] Pronin E., Lin D.Y., Ross L. (2002). The Bias Blind Spot: Perceptions of Bias in Self Versus Others. Personal. Soc. Psychol. Bull..

