# Supplementary material for: Healthcare Students and Medical Residents as Second Victims: A Cross-Sectional Study
Source: Int J Environ Res Public Health. 2022 Sep 26;19(19):12218. doi: 10.3390/ijerph191912218 (PMC9564455; doi:10.3390/ijerph191912218)
Supplement: Supplementary file 1 [file ijerph-19-12218-s001.zip › ijerph-1913789-supplementary.pdf]

Supplementay Materials S1 to *"Health Care students as second victims: a cross sectional study"* (Rinaldi et al.) - Complete Questionnarie

Code	Question	Possible Answers (if closed)
Q1	**Start Time**	
Q2	**End Time**	
Q3	**1.Età (Age)**	
Q4	**2.Genere (Gender)**	M / F
Q5	**3.Mesi di tirocinio (Months of training)**	
Q6	**Tirocinio aggiustato (Adjusted training months)**	
Q7	**4.Anno accademico di immatricolazione (academic year of enrolment)**	
Q8	**5.Corso di studio<br>(Typology of study course, with detail of residency programs)**	Infermieristica=1;M&Cmcu=2;SpecAnest&Rian=3;SpecIgi&Prev=4; SpecMeu=5;SpecMFR=6;SpecOrtop=7;SpecPedia=8;SpecGine=9;SpecRadio=10;SpecMI=11;SpecCardio=12;SPecPsic=13;SPecNeuro=14;SpecChir=15;SpecOtor=16
Q9	**5.Corso di studio<br>(Typology of study course, residency programs as aggregate category)**	Infermieristica/Nurse=1;M&C/Medicine=2;Spec/Resident=3
Q10	**6.Hai mai avuto esperienza o sei mai stato testimone di un PSI che ha avuto conseguenze su uno o più pazienti?<br>(Have you ever experienced a PSI with consequences on one or more patients?)**	1=No,non sono mai stato coinvolto; 2=Sì,dall'esterno; 3=Sì,ho effettuato io la manovra; 4=sì, ho partecipato alla manovra
Q11	**7.Che impatto ha avuto sul paziente il più severo PSI in cui sei stato direttamente coinvolto?<br>(How severe was the impact of the PSI you were involved in on the patient?)**	0=nessuna lesione;1=lesione temporanea; 2=lesione permanente; 3=morte del paziente
Q12	**8.In quanti PSI sei stato coinvolto negli ultimi 6 mesi?<br>(How many PSI have you been involved in during the last 6 months?)**	
Q13	**9.Che impatto ha avuto sul paziente il più severo PSI a cui hai assistito?<br>(What impact the most severe PSI you experienced had on the patient?)**	0=nessuna lesione;1=lesione temporanea; 2=lesione permanente; 3=morte del paziente
Q14	**10.A quanti PSI hai assistito negli ultimi 6 mesi?<br>(How many PSI have you been involved into during the last 6 months?)**	
Q15	**11.Quando è avvenuto il PSI o, in caso di più PSI, quello più significativo?<br>(When did the PSI or, in case of multiple PSIs, the most significant PSI happen?)**	1=1 mese fa;2=1-6 mesi fa; 3=6-12 mesi fa; 4=più di 1 anno fa
Q16	**12.In quale setting lavorativo è avvenuto questo PSI?<br>(What was the setting of the PSI?)**	1=ospedaliero; 2= casa di riposo; 3= altro(continuità ass)
Q17	**13.Potresti descrivere esattamente cosa è accaduto?<br>(Can you describe in detail what happened?)**	
Q18	**14(a). Quale pensi che sia stata la causa di questo PSI?: Distrazione<br>(In your opinion, what was the cause of this PSI? Distraction)**	0=niente a che vedere;1=molto a che fare; 2=non so
Q19	**14(b).Errore di comunicazione (Comunication error)**	0=niente a che vedere;1=molto a che fare; 2=non so
Q20	**14(c).Alta pressione lavorativa (High working pressure)**	0=niente a che vedere;1=molto a che fare; 2=non so
Q21	**14(d).Esperienza insufficiente (Insufficient/lacking experience)**	0=niente a che vedere;1=molto a che fare; 2=non so
Q22	**14(e).Conoscenza insufficiente (Insufficient/lacking Knowledge)**	0=niente a che vedere;1=molto a che fare; 2=non so
Q23	**14(f). Supervisione insufficiente (Insufficient/lacking supervision)**	0=niente a che vedere;1=molto a che fare; 2=non so
Q24	**14(g).Errore di procedura (Procedure error)**	0=niente a che vedere;1=molto a che fare; 2=non so
Q25	**15.Secondo la tua opinione il PSI si sarebbe potuto evitare?<br>(In your opinion, do you think this PSI could have been avoided?)**	0=evitabile; 1=molto facile da evitare; 2=facile da evitare; 3= difficile da evitare; 4=molto difficile da evitare
Q26	**16.Hai risentito del PSI? (Have the PSI had an impact on your life?)**	0=Non ha avuto impatto significativo; 1= Impatto vita profess; 2=impatto vita pers; 3= sì, profess e person
Q27	**17(a). Per quanto tempo hai sofferto delle seguenti sensazioni/emozioni negative come conseguenza del PSI? : Paura<br>(How long have you experienced this negative emotion/feeling as a consequence of the PSI? Fear)**	0=mai; 1=poche ore; 2=1gg; 3=<1 settimana; 4=<1mese; 5= 1-6 mesi; 6= 7-12 mesi;7=>1anno

Q28	**17(b).Flashback (Flashback)**	0=mai; 1=poche ore; 2=1gg; 3=<1 settimana; 4=<1mese; 5= 1-6 mesi; 6= 7-12 mesi;7=>1anno
Q29	**17(c).Ipervigilanza (Hypervigilance)**	0=mai; 1=poche ore; 2=1gg; 3=<1 settimana; 4=<1mese; 5= 1-6 mesi; 6= 7-12 mesi;7=>1anno
Q30	**17(d).Sensazione di lavorare male (Feeling of working badly)**	0=mai; 1=poche ore; 2=1gg; 3=<1 settimana; 4=<1mese; 5= 1-6 mesi; 6= 7-12 mesi;7=>1anno
Q31	**17(e).Infelicità e depressione (Unhappiness / depression)**	0=mai; 1=poche ore; 2=1gg; 3=<1 settimana; 4=<1mese; 5= 1-6 mesi; 6= 7-12 mesi;7=>1anno
Q32	**17(f).Disagio nel team di lavoro (Uneasiness in working team)**	0=mai; 1=poche ore; 2=1gg; 3=<1 settimana; 4=<1mese; 5= 1-6 mesi; 6= 7-12 mesi;7=>1anno
Q33	**17(g).Vergogna, senso di colpa (Shame, sense of guilt)**	0=mai; 1=poche ore; 2=1gg; 3=<1 settimana; 4=<1mese; 5= 1-6 mesi; 6= 7-12 mesi;7=>1anno
Q34	**17(h).Insonnia (Insomnia)**	0=mai; 1=poche ore; 2=1gg; 3=<1 settimana; 4=<1mese; 5= 1-6 mesi; 6= 7-12 mesi;7=>1anno
Q35	**17(i). Stress (Stress)**	0=mai; 1=poche ore; 2=1gg; 3=<1 settimana; 4=<1mese; 5= 1-6 mesi; 6= 7-12 mesi;7=>1anno
Q36	**17(j). Dubbi circa le proprie conoscenze e abilità (Doubts about own skills and knowledge)**	
Q37	**18.Hai pensato di abbandonare gli studi dopo questo PSI? (Have you ever considered quitting the study course after the PSI?)**	1=no;2=sì
Q38	**19.Hai colto qualche elemento positivo in questa esperienza? (Did you identify any positive element from this experience?)**	1=no;2=sì
Q39	**20.Potresti descrivere quale elemento positivo hai colto in questa esperienza? (Can you describe in detail any positive element identified?)**	
Q40	**21(a). Hai mai parlato con qualcuno di questo incidente?: Tutor clinico (Have you ever spoken to anyone about this incident? Clinical tutor)**	1=sì, esperienza positiva;2=sì,esperienza negativa; 3=no ma mi dispiace; 4=no, ma è stata la scelta giusta
Q41	**21(b). Tutor didattico (Didactic tutor)**	1=sì, esperienza positiva;2=sì,esperienza negativa; 3=no ma mi dispiace; 4=no, ma è stata la scelta giusta
Q42	**21(c). Docente/Professore (Professor)**	1=sì, esperienza positiva;2=sì,esperienza negativa; 3=no ma mi dispiace; 4=no, ma è stata la scelta giusta
Q43	**21(d). Counseling dell'Università (University counseling service)**	1=sì, esperienza positiva;2=sì,esperienza negativa; 3=no ma mi dispiace; 4=no, ma è stata la scelta giusta
Q44	**21(e). Colleghi di università (University collegues)**	1=sì, esperienza positiva;2=sì,esperienza negativa; 3=no ma mi dispiace; 4=no, ma è stata la scelta giusta
Q45	**21(f). Infermieri (Nurses)**	1=sì, esperienza positiva;2=sì,esperienza negativa; 3=no ma mi dispiace; 4=no, ma è stata la scelta giusta
Q46	**21(g). Coordinatore infermieristico (Head nurse)**	1=sì, esperienza positiva;2=sì,esperienza negativa; 3=no ma mi dispiace; 4=no, ma è stata la scelta giusta
Q47	**21(h). Medici (Physicians)**	1=sì, esperienza positiva;2=sì,esperienza negativa; 3=no ma mi dispiace; 4=no, ma è stata la scelta giusta
Q48	**21(i). Medico di famiglia (Family doctor)**	1=sì, esperienza positiva;2=sì,esperienza negativa; 3=no ma mi dispiace; 4=no, ma è stata la scelta giusta
Q49	**21(j). Famigliari (Relatives)**	1=sì, esperienza positiva;2=sì,esperienza negativa; 3=no ma mi dispiace; 4=no, ma è stata la scelta giusta

Q50	**21(k). Amici (Friends)**	1=sì, esperienza positiva;2=sì,esperienza negativa; 3=no ma mi dispiace; 4=no, ma è stata la scelta giusta
Q51	**21(l). Partner (Partner)**	1=sì, esperienza positiva;2=sì,esperienza negativa; 3=no ma mi dispiace; 4=no, ma è stata la scelta giusta
Q52	**21(m). Paziente (Patient)**	1=sì, esperienza positiva;2=sì,esperienza negativa; 3=no ma mi dispiace; 4=no, ma è stata la scelta giusta
Q53	**21(n). Familiari del paziente (Patient relatives)**	1=sì, esperienza positiva;2=sì,esperienza negativa; 3=no ma mi dispiace; 4=no, ma è stata la scelta giusta
Q54	**22.Se ne hai parlato con il paziente e/o i suoi familiari, come hai affrontato questa conversazione? (If you spoke to the patient and/or his/her relatives, how did you conduct such conversation?)**	
Q55	**23.Se ne hai parlato con il paziente e/o i suoi familiari, quale esperienza hai tratto da questa conversazione? (If you spoke to the patient and/or his/her relatives, what lesson have you learned from this conversation?)**	
Q56	**24(a). Se fossi mai coinvolto in un PSI nel futuro, con chi ne parlersti?: Tutor clinico (If you were ever involved in a PSI in the future, who would you talk to about it? Clinical Tutor)**	1=sì; 2=no; 3=non so
Q57	**24(b). Tutor didattico (Didactic tutor)**	1=sì; 2=no; 3=non so
Q58	**24(c). Docente/Professore (Professor)**	1=sì; 2=no; 3=non so
Q59	**24(d). Counseling dell'Università (University counseling service)**	1=sì; 2=no; 3=non so
Q60	**24(e). Colleghi di università (University collegues)**	1=sì; 2=no; 3=non so
Q61	**24(f). Infermieri (Nurses)**	1=sì; 2=no; 3=non so
Q62	**24(g). Coordinatore infermieristico (Head nurse)**	1=sì; 2=no; 3=non so
Q63	**24(h). Medici (Physicians)**	1=sì; 2=no; 3=non so
Q64	**24(i). Medico di famiglia (Family doctor)**	1=sì; 2=no; 3=non so
Q65	**24(j). Famigliari (Relatives)**	1=sì; 2=no; 3=non so
Q66	**24(k). Amici (Friends)**	1=sì; 2=no; 3=non so
Q67	**24(l). Partner (Partner)**	1=sì; 2=no; 3=non so
Q68	**24(m). Paziente (Patient)**	1=sì; 2=no; 3=non so
Q69	**24(n). Familiari del paziente (Patient relatives)**	1=sì; 2=no; 3=non so
Q70	**25(a). Cosa faresti se un paziente e la sua famiglia volessero parlare con te dopo un PSI?: Vorrei scusarmi (What would you do if a patient and their family wanted to talk to you after a PSI ?: I would like to apologize)**	1=sì; 2=no; 3=non so
Q71	**25(b). Ascolterei e spiegherei tutto nel modo migliore possibile (I would listen and explain everything in the best possible way)**	1=sì; 2=no; 3=non so
Q72	**25(c). Ascolterei e spiegherei solo il minimo (I would listen and explain with minimal details)**	1=sì; 2=no; 3=non so
Q73	**25(d).Sosterrei la discussione ma non aggiungerei nient'altro (I would sustain the discussion but not adding any detail)**	1=sì; 2=no; 3=non so
Q74	**25(e). Eviterei ogni dialogo (I would avoid any conversation)**	1=sì; 2=no; 3=non so
Q75	**26(a). Supponiamo che nel futuro tu fossi coinvolto in un PSI. Cosa faresti?: Porrei più attenzione ai dettagli del mio lavoro (Let's assume that in the future you were involved in a PSI. What would you do ?: I would like to pay more attention to the details of my work)**	1=sì; 2=no; 3=non so
Q76	**26(b). Ne parlerei il meno possibile (I would talk about it as little as possible)**	1=sì; 2=no; 3=non so
Q77	**26(c).Cambierei il mio modo di lavoro al fine di non ripetere l'errore (I would change my way of working in order not to repeat the mistake)**	1=sì; 2=no; 3=non so
Q78	**26(d). Starei lontano da pazienti e situazioni analoghe (I would stay away from patients and similar situations)**	1=sì; 2=no; 3=non so

Q79	**26(e). Chiederei ad un collega che cosa avrebbe fatto in un caso simile (I would ask a colleague what he would have done in a similar case)**	1=sì; 2=no; 3=non so
Q80	**26(f).Chiederei se i pazienti sono soddisfatti del mio operato (I would ask if the patients are satisfied with my work)**	1=sì; 2=no; 3=non so
Q81	**27.Quanto pensi sia probabile essere coinvolti in un PSI? (How often do you think it is probable to be involved in a PSI?)**	1=mensilmente;2=1/anno; 3=<1/anno;4=rari
Q82	**28.Sai di altri tuoi colleghi che sono stati coinvolti in un PSI? (Do you have any notice of collegues involved in a PSI?)**	
Q83	**28(b).Se sì, quanti? (If yes, how many?)**	
Q84	**29.Ti sentiresti di dare qualche consiglio alla tua Università? (Do you want to make any suggestion to your University?)**	
Q85	**30.Telefono (Phone number)**	
Q86	**31.E-mail**	
